# The Principles and Practice of Medicine, in a Series of Essays

**Published:** 1848-10

**Authors:** 


*The Principles and Practice of Medicine, in a series of Essays.* By John W. Hood, M. D. 8vo. pp. 263. Thomas, Cowperthwaite & Co. Philadelphia: 1848.

To those who think a great book is a great evil, it will be good news to hear of the Principles and Practice of Medicine being reduced within the compass of 263 octavo pages, well leaded, and with good wide margins, which render a book so agreeable to old eyes, as well as satisfactory to the furtive glances of the young. The book is a new one, too. The author's opinions of pathology and therapeutics are really original, and we believe not to be found in any preceding book, which must delight modern reviewers, who have been wont to prove that there is nothing new under the sun, unless it chance to be their own lucubrations, or the wise cogitations or discoveries of a particular friend, or unless it emanates from a particular district. Practical people, too, will be pleased; for it is the offspring of the author's own experience—albeit, a thread of theory runs through the whole, and binds together the several parts.

"Each author," says Dr. Hood, "if possible, should at least endeavour to rank with those of the preceding age, and it would be better for the profession if each contributor would give only what he has, or believes he has discovered, without borrowing, curtailing, or extracting opinions already published." It appears to us that Dr. H. has adhered to his own rule with much more fidelity than the generality of authors and legislators; for, except some of his own previously published opinions, the book certainly contains marvellously few of the discoveries and opinions to be found in books, ancient or modern.

The Essays which constitute this volume of "THE PRINCIPLES AND PRACTICE OF MEDICINE," treat of the "*Anatomical arrangement and configuration of the Body; Chronic diseases of the Viscera of the Abdomen; Strumous Habit; Fever; Displacement of the Womb; Asiatic Cholera; Mechanical Agents; Reducible Hernia; Hemorrhoids, or Piles; Gout; the Tongue;*" and an "*Appendix*" of six pages.

If this enumeration of subjects falls short of what we are accustomed to meet with in works with so comprehensive a title, it at least includes a very good *variety.*

We have not had time to read this modern production through, and are therefore not qualified to give an impartial opinion upon its merits; but to afford the reader some opportunity of judging for himself of the author's *fundamental* doctrine, we quote the following from page 27, on the "Anatomical arrangement and configuration of the Body." As far as we can judge, it embraces the *corner stone* of the grand superstructure.

"From a knowledge of the laws which govern the organs, and enable them to perform their specific functions, is it not strange that the various writers should have overlooked the cavity of the abdomen with its important assemblage of organs? all of which are dependent upon the voluntary or involuntary movements of the nine muscles, and the connection with the serratus and latissimus dorsi. Sir Charles Bell, in his researches on the nervous system, which presides over the respiratory muscles, conclusively assigns the functions, and why is it that the physiologist has not been called to the mechanical functions of this part of myology? The failure of these muscles invariably leads to the destruction of health, and eventuates more frequently in death, than from all other causes—yet it is not to be understood that the failure is the remote cause in all, but it becomes an exciting one in some, and an irritating contributor to every functional or constitutional disease. In the debilitated or exhausted power of those muscles, it has been already shown, the viscera and fluids gravitate, and by the mechanical functions, with the assistance of the intercostal muscles, diaphragm and mobility of the ribs, inspiration and expiration are performed. Hence is it not evident and demonstrable that the lungs in their hinder and lower portions fail to dilate, and be traversed by the air? On such failures, which leave the blood in a pathological condition, is it not clear that the lower portion of the lungs becomes the seat of fatal congestions? Hence the importance of mechanical agents to assist the organs in this debilitated condition, until their lost energy is restored."

As the reader may be curious to know something of the author's principles of diagnosis, we subjoin another extract:

"The failure of the organs of the abdomen is easily detected by a careful examination of the physiognomy of the patient. The strength of the body and the state of the mind, with the color and condition of the skin, are indexes of prevailing malady. But having thus ascertained the condition as far as sight enables us to determine it, we next make a careful exploration of the chest, by percussion and auscultation. After which sight and touch is combined, and having determined by the first the configuration, we trace by the second the organs that are liable to displacement, and ascertain, if possible, their size and position. Such examination is absolutely necessary in cases where the predisposing or exciting cause is suspected to have originated in the gravitation of the solids and fluids; without such examination our prognosis must more frequently destroy the confidence of the patient than otherwise. The importance of the organs of those two cavities, demands the closest investigation, and if we expect to sustain the dignity of our profession, we must do it by mitigating or curing the afflictions of the human family. The want of such attention is already manifest in the lost confidence of the public, and in the rapid growth of new systems which may be ascribed to the inattention of the faculty, and the credulity of the public. But if we discover that the difficulty arises from hereditary taint, and the appearances are not of a character to define the cause, the habits of the patient, the age, sex, constitution, idiosyncrasy if any, and occupation, will enable us to judge correctly, and apply the agents adapted to the condition of the patient."

